# The Principles and Practice of Obstetrics

**Published:** 1862-05

**Authors:** 


### Book Notices.

THE PRINCIPLES AND PRACTICE OF OBSTETRICS. By GUNNING S. BEDFORD, A.M., M.D., Prof. of Obstetrics and of Diseases of Women and Children, and of Clinical Obstetrics, in the University of New York; Author of "Clinical Lectures on Diseases of Women and Children." Second Edition. New York: William Wood, 389 Broadway.

As the first edition of this work was issued scarcely six months since, and was then highly recommended in the pages of the EXAMINER, it is unnecessary to make any extended notice of this second edition. Indeed, the fact that a second edition has been called for in so short a time, is the strongest proof of the high estimation placed upon it by the profession. It is a very full and complete treatise on the Science and Art of Obstetrics. The publishers have also executed their part of the work in good style. It should have a place in the library of every practitioner.

